# A bidirectional Mendelian randomisation study to evaluate the relationship between body constitution and hearing loss

**DOI:** 10.1038/s41598-023-44735-x

**Published:** 2023-10-27

**Authors:** Yiyan He, Ville Karhunen, Anna Pulakka, Marko Kantomaa, Sylvain Sebert

**Affiliations:** 1 Research Unit of Population Health, Faculty of Medicine, University of Oulu, Oulu, Finland; 2 Research Unit of Mathematical Sciences, Faculty of Science, University of Oulu, Oulu, Finland; 3 Population Health Unit, Finnish Institute for Health and Welfare, Helsinki, Finland

**Keywords:** Epidemiology, Genetics

## Abstract

Hearing loss and hearing disorders represent possible mediating pathways in the associations between noise exposures and non-auditory health outcomes. In this context, we assessed whether the noise-obesity associations should consider hearing functions as possible mediators and applied Mendelian randomisation (MR) to investigate causal relationships between body constitution and hearing impairments. We obtained genetic associations from publicly available summary statistics from genome-wide association studies in European ancestry adult populations (N= from 210,088 to 360,564) for (i) body constitution: body mass index (BMI), waist circumference (WC) and body fat percentage (BFP), and (ii) hearing loss: sensorineural hearing loss, noise-induced hearing loss, and age-related hearing impairment (ARHI). We employed colocalisation analysis to investigate the genetic associations for BMI and ARHI liability within an *FTO* locus. We conducted bi-directional MR for the ‘forward’ (from body constitution to hearing) and ‘reverse’ directions. We applied the random effects inverse variance-weighted method as the main MR method, with additional sensitivity analyses. Colocalisation analysis suggested that BMI and ARHI shared a causal variant at the *FTO* gene. We did not find robust evidence for causal associations from body constitution to hearing loss and suggested that some associations may be driven by *FTO* variants. In the reverse analyses, ARHI was negatively associated with BMI [effect size – 0.22 (95% CI – 0.44 to – 0.01)] and BFP [effect size – 0.23 (95% CI – 0.45 to 0.00)], supporting the notion that ARHI may diminish body constitution. Finally, our data suggest that there is no strong evidence that hearing explains the association between noise exposure and body constitution.

## Introduction

There is evidence from observational studies that long-term exposure to traffic noise is associated with an increasing risk of obesity^1^. However, the WHO environmental noise guidelines recently rated the research quality on environmental noise exposure and obesity low and emphasised the importance of studying hearing-related outcomes associated with noise exposure^2^. Hearing loss is indeed a growing health concern that may mediate or confound the association between noise exposure and its attributed non-auditory health outcomes^3^.

More frequently studied, hearing loss has been shown to be positively associated with excess weight^4^ and its related cardiometabolic comorbidities, such as cardiovascular disease^5,6^ and diabetes mellitus^7^. The association is biologically plausible since people with these metabolic diseases have a high probability for poor micro-vascular circulation that reduces blood supply to the cochlea, resulting in damage to the hair cells and, eventually, sensorineural hearing loss. Additional studies show consistent results that elevated body mass index (BMI) is positively associated with hearing loss^8–10^. A recent meta-analysis systematically reviewed the 14 published observational studies and found a positive association between hearing loss and BMI and waist circumference (WC)^11^.

However, the association between body constitution and hearing loss remain inconclusive, as other studies found no, or even a negative association between both conditions^10^. Amid this uncertainty, some studies found that low BMI was also positively associated with hearing loss^12^. This association was further supported by a recent large-scale Korean population study, which reported that lower BMI (< 18.5 kg/m^2^) was associated with an increased risk of hearing loss^13^. The associations were stronger in men and old people than in women and young generations. Furthermore, an earlier study suggested that malnutrition and being underweight are positively associated with sensorineural hearing loss^14^, which could be partly a result of degeneration and demyelination of the eight cranial nerve and mitochondrial impairment-affecting neurons.

In the previous literature, the mechanism of the associations was mostly discussed from body constitution to hearing loss and the direction of inference of the two phenotypes lacks investigation from hearing loss to body constitution. The previous studies were observational and cannot rule out the questions of reverse causation, selection bias, survival bias and residual confounding. Conversely, the Mendelian randomisation (MR) approach is more robust to unmeasured confounding and reverse causation which hamper accurate causal inference in conventional epidemiological research.

To address these objectives, we set up a genetically informed study (MR) to infer the potential causality and its direction between body constitution and hearing loss. It is plausible that both the forward (from body constitution to hearing loss) and the reverse (from hearing loss to body constitution) association can exist and therefore we hypothesised a bi-directional causation.

## Methods

We conducted a two-sample, bi-directional MR. Genetic associations for all traits were obtained from publicly available summary statistics of genome-wide association studies (GWAS) (Table 1). The data were restricted to European populations. To avoid potential weak instrument bias due to sample overlap^15^, we sought GWAS with non-overlapping samples whenever possible. This was not possible for the analyses between age-related hearing impairment (ARHI) and body fat percentage (BFP), where we used the GWAS of UK Biobank with a maximum 70% participant overlap for these two phenotypes. Since the genetic associations of both BMI and ARHI have a strong signal within the *FTO* gene region, we conducted colocalisation analysis before the MR analysis to examine whether this signal is driven by a shared causal variant, or whether the association is due to confounding by linkage disequilibrium (LD).

**Table 1:** Sources of secondary summary statistics used to derive instrumental variables for adult body constitution and hearing loss.

Phenotypes	Source	Year of publication	Codes^*^, references or ICD codes	Sample size	Cases (n)	Controls (n)	Cases (%)
Body constitution-related phenotypes							
Body mass index	UK Biobank	2018	21001	359,983	NA	NA	NA
Body mass index	GIANT	2015	Locke et al.^16^	322,154	NA	NA	NA
Waist circumference	UK Biobank	2018	48	360,564	NA	NA	NA
Waist circumference	GIANT	2015	Shungin et al.^17^	210,088	NA	NA	NA
Body fat percentage	UK Biobank	2018	23099	354,628	NA	NA	NA
Hearing loss-related phenotypes							
Sensorineural hearing loss	FinnGen	Release 6, 2022	ICD 10: H90.3-5; ICD9: 3891	252,719	19,313	233,406	7.64
Noise-induced hearing loss	Finngen	Release 6, 2022	ICD 10: H83.3; ICD 9: 3881	249,936	655	249,281	0.26
Age-related hearing impairment	UK Biobank	2019	Wells et al.^19^	250,389	87,056	163,333	34.77

*GIANT* Genetic Investigation of ANthropometric Traits, *ICD* International Classification of Disease. * UK Biobank phenotype ID codes

### Data sources for body constitution-related phenotypes

The adult body constitution was measured by BMI (kg/m^2^), WC (cm) and BFP (%). For BMI and WC, we used sex-combined joint GWAS and Metabochip meta-analysis from the Genetic Investigation of ANthropometric Traits (GIANT) involving 114 studies and 322,154 participants (mean age: 55.52 years, 46.02% men, mean BMI: 27.40 kg/m^2^) reported by Locke^16^, and 101 studies and 210,088 participants (mean age: 55.25 years, 45.39% men, mean WC: 93.07 cm) reported by Shungin^17^, respectively. Both GWAS were used in the MR analyses with ARHI from UK Biobank.

Apart from GIANT, the data on BMI (n= 359,983) and WC (n= 360,564) were additionally obtained from UK Biobank^18^. The two traits were assessed by anthropometric measurements. We also retrieved the GWAS of BFP from UK Biobank with 354,628 individuals. All traits used here from UK Biobank are continuous phenotypes that have been measured during the initial assessment visit (2006–2010) and rank normalised. The descriptions of BMI, WC and BFP from UK Biobank (data fields: 21001, 48 and 23099, respectively) were presented on its webpage (https://biobank.ctsu.ox.ac.uk/crystal/search.cgi).

### Data sources for hearing loss-related phenotypes

Hearing loss traits included sensorineural hearing loss (SNHL), noise-induced hearing loss (NIHL) and ARHI. GWAS on risk of ARHI (87,056 cases and 163,333 controls, mean age: 60 years) was from Wells et al.^19^ based on a self-reported hearing difficulty (*HDiff*) phenotype in the UK Biobank. The details of the questionnaires and case-control assignments for the *HDiff* phenotype can be found in their published supplement material.

Summary-level genetic data for risk of SNHL and NIHL were obtained from the FinnGen^20^ release 6 including 271,341 participants. The phenotypes were collected from nationwide hospital discharge and cause of death registers using the International Classification of Diseases (ICD) codes for SNHL: H90.3, H90.4, H90.5 (ICD-10) and 3891 (ICD-9) and NIHL: H83.3 (ICD-10) and 3881 (ICD-9) (Table 1). Genetic association estimates for SNHL liability were obtained from 19,313 cases (mean age: 63.37 years, 51.72% men) and 233,406 controls, and for NIHL liability from 655 cases (mean age: 52.43 years, 92.82% men) and 249,281 controls.

### Genetic instrumental variables

Whenever possible, the instrumental variables (IVs) for the exposures were composed of genome-wide significant (P<5×10^−8^) single-nucleotide polymorphisms (SNPs) that are associated with the corresponding phenotypes in the aforementioned GWAS. This included 12 SNPs for SNHL and none for NIHL. Therefore, to enable a sufficient number of IVs for MR sensitivity analyses, we included SNPs with P-value <5×10^−5^ as instruments for these traits^21^.

We harmonised the effect alleles in the exposure and outcome datasets based on the guidelines provided by Hartwig^22^ and excluded palindromic variants with minor allele frequency (MAF)>0.4 and imputation info score<0.9. We selected independent SNPs (LD) R^2^≤0.001 within a 10000 kb window using the ‘ld_clump’ function on the mrcieu/ieugwasr package. If a variant was unavailable in the outcome GWAS summary statistics, then proxy SNPs with a minimum LD R^2^=0.9 were searched for.

F and R^2^ statistics were calculated for the individual IVs to quantify instrument strength using the method described by Burgess et al.^15^. The statistical power for continuous exposures was evaluated by calculating the approximate minimum detectable odds ratio (OR) for each exposure at a power of 80%, given the sample size of the exposure, the total variance explained by the instruments and the type 1 error rate of 0.05.

### Colocalisation analysis

Variants at the *FTO* locus were associated with BMI and ARHI but it was unclear whether both phenotypes share the same causal variant. We conducted colocalisation analysis to examine it with the method ‘coloc’ developed by Giambartolomei et al.^23^, assuming a maximum of one causal variant per genomic locus. The method outputs posterior probabilities (PP) for five models of (H_0_) no causal variants, (H_1_) causal variant for exposure, (H_2_) causal variant for outcome, (H_3_) distinct causal variants for exposure and outcome, and (H_4_) shared causal variant for exposure and outcome. A PP>0.8 for model H_4_ was considered evidence of colocalization^23^. We examined the variants within the *FTO* gene locus (chr16: 53,737,875–54,155,853 on hg19) and used the default prior probabilities of a variant associated with BMI only (P_1_) and ARHI only (P_2_) at 10^−4^, and a variant associated with both traits (P_12_) at 10^–5^.

### Statistical analyses

We used the inverse variance weighted (IVW) method as the primary analysis in both forward and reverse MR to calculate the IVW estimator by meta-analysing the SNP-specific Wald estimates using a multiplicative random effects model. The Wald estimate for each SNP was calculated as a ratio between the coefficients for the SNP-to-outcome and the SNP-to-exposure regressions presented in the summary statistics^24^. The random effects model was chosen to account for potential heterogeneity, evaluated by Cochran's Q (assuming balanced pleiotropy)^25^. Evidence of heterogeneity was defined with a P-value < 0.05 in the Cochran's Q. We used R version 4.2.0^26^ and TwosampleMR package^27^ for all analyses.

### Sensitivity analyses

There are three assumptions to consider before validating MR Wald ratios as a robust inference of causation: the IVs (i) are associated with the exposure, (ii) are independent of the confounders and (iii) have no effects on outcome other than via the exposure. In particular, horizontal pleiotropy, in which the IVs have an effect on other traits outside of the pathway of the exposure of interest and have an impact on the outcome of interest, or when the IVs have a direct effect on the outcome of interest, violates the third assumption^28^. We adopted complementary MR methods that make different assumptions about horizontal pleiotropy to assess the robustness of the main MR findings: MR-Egger^29^, weighted median^30^, weighted mode^31^ and MR-PRESSO (Mendelian Randomisation Pleiotropy RESidual Sum and Outlier). MR-PRESSO was also used to detect outliers that may indicate pleiotropic effects^28^. The following additional analyses were conducted: single SNP analysis and leave-one-out analysis to provide visualisation and interpretation of two-sample MR^32^.

### Ethical approval

The present research used publicly available summary data and did not contact participants, where no extra ethical approval is required.

## Results

### Instrumental variable characteristics

Table 2 provided the number of IVs used and the minimal detectable odds ratios (ORs) for the forward analysis. Given the lack of individual-level data access, it was not possible to provide the detectable effect sizes for binary exposures in the reverse analyses. The association estimates for the IVs and their individual F statistics were presented in the Supplementary spreadsheets. There were a greater number of IVs for the body constitution phenotypes in the forward analyses than for hearing loss phenotypes in the reverse analyses. F statistics ranged between 52 and 67 in forward MR and between 19 and 42 in reverse MR, all of which were greater than 10, indicating little possibility of weak instrument bias (Supplementary Table 1).

**Table 2:** Summary data and statistical power calculation for the forward analyses.

Exposures	Source	Outcomes	Source	Included Variants (n)	Variance explained (%)	Detectable OR*
BMI (kg/m^2^)	UKB	SNHL	FinnGen	89	1.51	1.19
BMI (kg/m^2^)	UKB	NIHL	FinnGen	89	1.51	2.44
BMI (kg/m^2^)	GIANT	ARHI	UKB	68	2.25	1.08
WC (cm)	UKB	SNHL	FinnGen	72	0.83	1.26
WC (cm)	UKB	NIHL	FinnGen	72	0.83	3.33
WC (cm)	GIANT	ARHI	UKB	41	1.42	1.10
BFP (%)	UKB	SNHL	FinnGen	85	0.71	1.28
BFP (%)	UKB	NIHL	FinnGen	85	0.71	3.67
BFP (%)	UKB	ARHI	UKB	87	0.73	1.15

*Minimum detectable odds ratio per 1 SD change in the exposure, at 80% power and type 1 error rate=0.05.

*BMI* body mass index, *WC* waist circumference, *BFP* body fat percentage, *SNHL* sensorineural hearing loss, *NIHL* noise-induced hearing loss, *ARHI* age-related hearing impairment, *UKB* UK Biobank, *GIANT* Genetic Investigation of ANthropometric Traits.

### Colocalisation analysis

**Figure 1:**
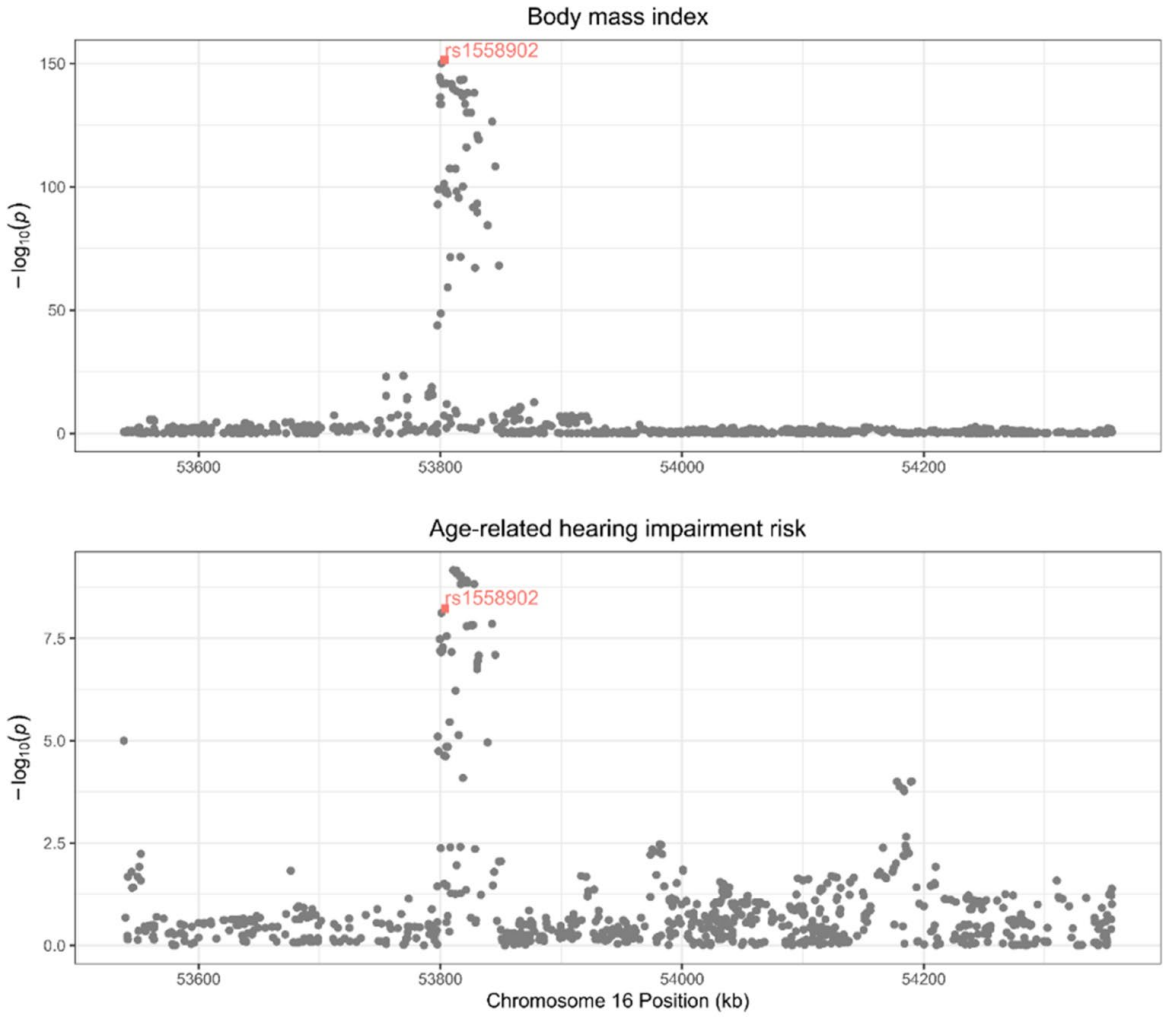
Regional association plots at the *FTO* locus for body mass index (BMI) and age-related hearing impairment (ARHI) risk. The hit single nucleotide polymorphisms (SNP) rs1558902 was marked in a coloured square shape. The regional plots have been drawn for the associations reported in the GIANT consortium and the UK biobank, respectively, for BMI and ARHI.

The results of the colocalisation analysis between BMI and ARHI were shown in Fig. 1 and the supplementary Table 2. We found a PP for H_4_ of 0.92, supporting a shared causal variant (rs1558902) for BMI and ARHI at the *FTO* locus. The allele A for variant rs1558902 was positively associated with BMI and negatively associated with ARHI and the MR estimate of BMI on ARHI using only this variant was 0.91 (95% confidence interval [CI]: 0.88 to 0.94). The variant was in perfect LD with *FTO* variant rs1421085 adopted in the forward MR analysis (LD R^2^ = 1). Therefore, based on the known effect of the *FTO* gene on BMI^33^, the *FTO* variant rs7193144 was excluded from the IVs of ARHI in reverse MR to avoid potential exposure misspecification.

### Forward analyses (from body constitution to hearing)

**Figure 2:**
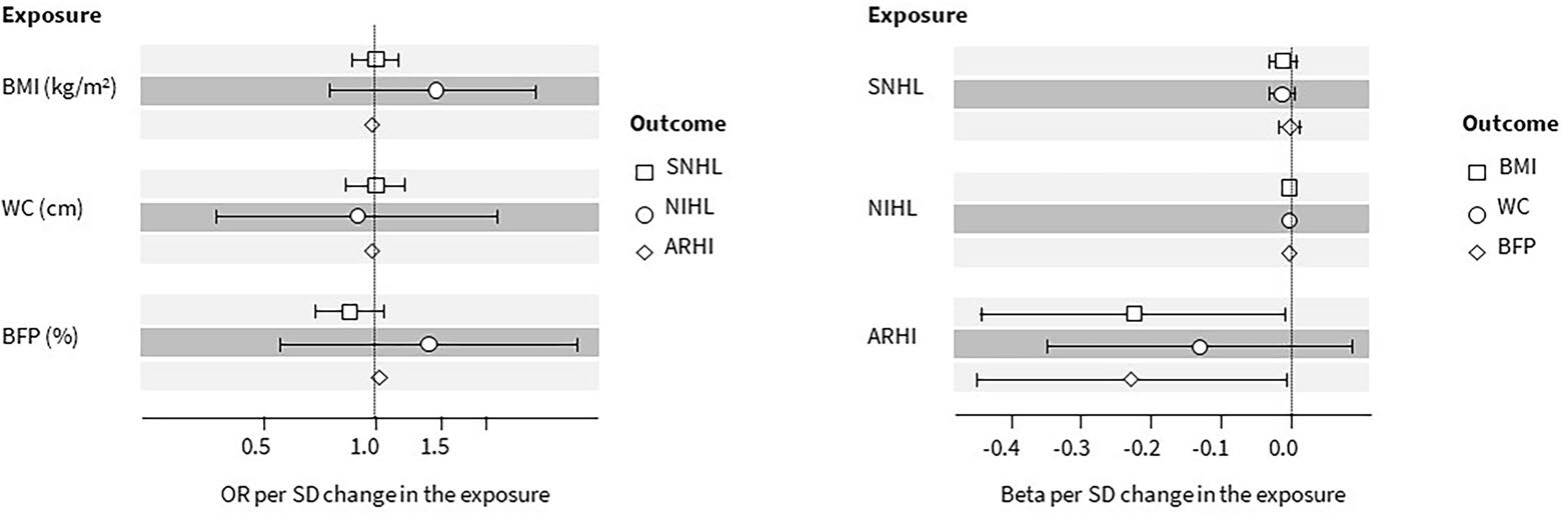
Forest plots for the causal estimates [odds ratios (OR) or beta coefficient and their 95% confidence intervals] showing the Wald ratio estimates for the association between body constitution [body mass index (BMI), waist circumference (WC) and body fat percentage (BFP)] and the risk of hearing loss [sensorineural hearing loss (SNHL), noise-induced hearing loss (NIHL), and age-related hearing impairment (ARHI)] (left panel), in the forward two-sample Mendelian randomisation (MR), and, between hearing loss and body constitution (right panel), in the reverse two-sample MR.

**Figure 3:**
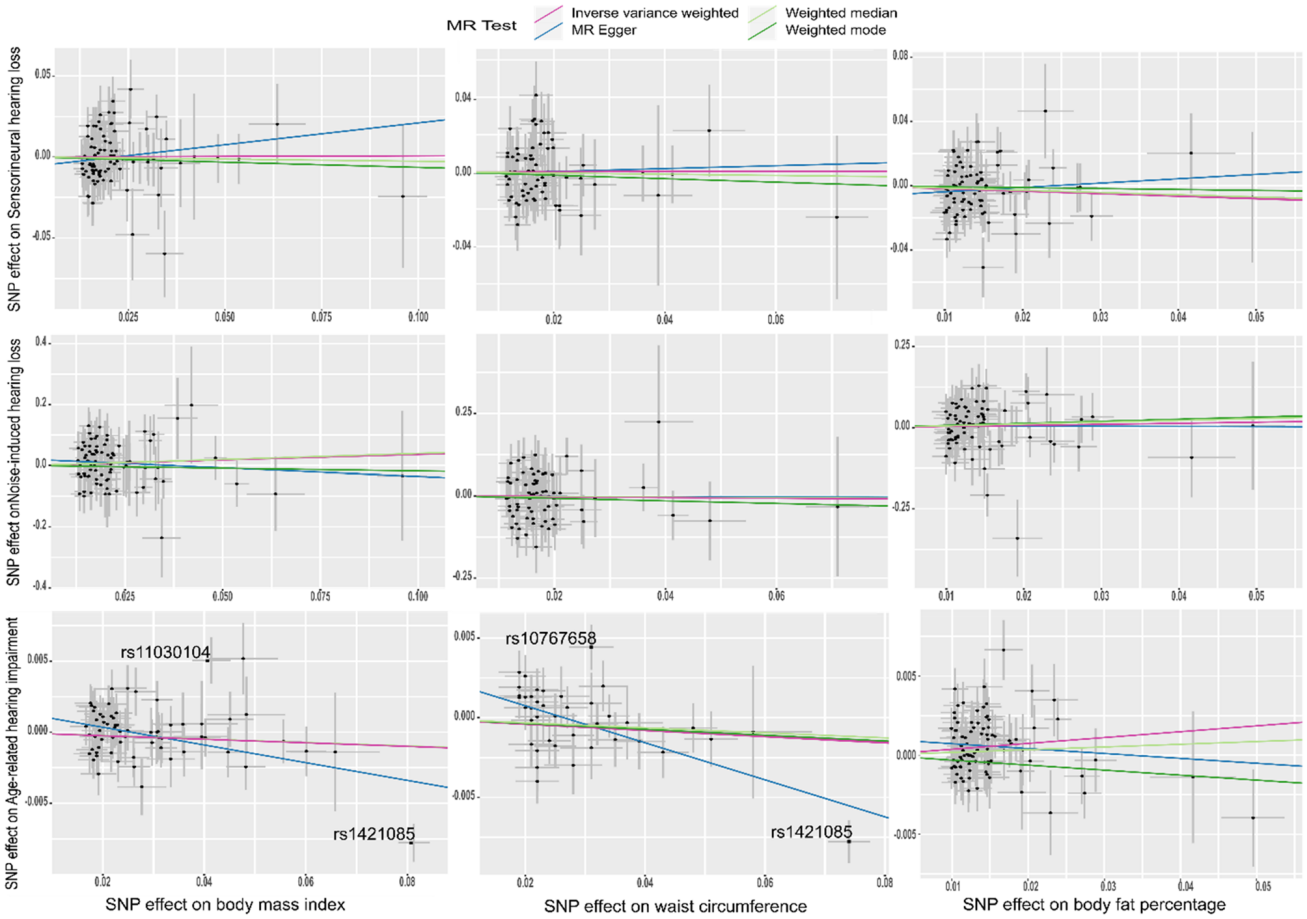
Scatter plots showing Mendelian randomisation sensitivity analysis estimates for the forward two-sample Mendelian randomisation. *MR-Egger* Mendelian randomisation Egger. Outlier instrumental variables (IVs) detected by MR-PRESSO are labelled with the rsid.

The results of the analyses were shown in Fig. 2 and the supplementary Table 3. According to our results, there was no evidence to support the notion of genetically predicted BMI being associated with any of the hearing loss outcomes included in these analyses. The resulting IVW odds ratios (ORs) per 1-SD increase in exposure were 1.01 (95% CI 0.88 to 1.16), 1.44 (95% CI 0.76 to 2.73) and 0.99 (95% CI 0.97 to 1.00) for risk of SNHL, NIHL and ARHI, respectively. Similarly, we found no evidence that genetically predicted WC was associated with hearing loss outcomes. The resulting IVW ORs were 1.00 (95% CI 0.84 to 1.20), 0.89 (95% CI 0.37 to 2.15) and 0.98 (95% CI 0.96 to 1.00) for SNHL, NIHL and ARHI, respectively. Genetically predicted BFP was not robustly associated with SNHL [OR 0.85 (95% CI 0.69 to 1.06)] or NIHL [OR 1.44 (95% CI 0.76 to 2.73)]. Finally, we observed that genetically predicted BFP was positively associated with the risk of ARHI [OR 1.04 (95% CI 1.01 to 1.06)]. However, the sensitivity analyses were not concordant with the IVW (Fig. 3, Supplementary Fig. 1 and Supplementary Table 4).

### Reverse MR

The results of the analyses were presented in Fig. 2 and the supplementary Table 5. Based on our results, there was no evidence to support the notion that genetically proxied SNHL was associated with any of the body constitution outcomes included in these analyses. The resulting IVW effect sizes for BMI, WC and BFP were 0.00 (95% CI – 0.03 to – 0.01), – 0.01 (95% CI – 0.03 to – 0.01) and 0.00 (95% CI – 0.01 to – 0.01), respectively. Similarly, we found no evidence that genetically predicted NIHL was associated with body constitution outcomes. There was some evidence of genetically proxied ARHI liability being associated with lower BMI and BFP. The observed effect sizes were – 0.22 (95% CI – 0.44 to – 0.01) and – 0.23 (95% CI – 0.45 to 0.00), respectively. Although we did not find evidence to support the association between ARHI liability and WC, the direction of the IVW effect size [– 0.13 (95% CI – 0.35 to 0.09)] was consistent with BMI and BFP.

**Figure 4:**
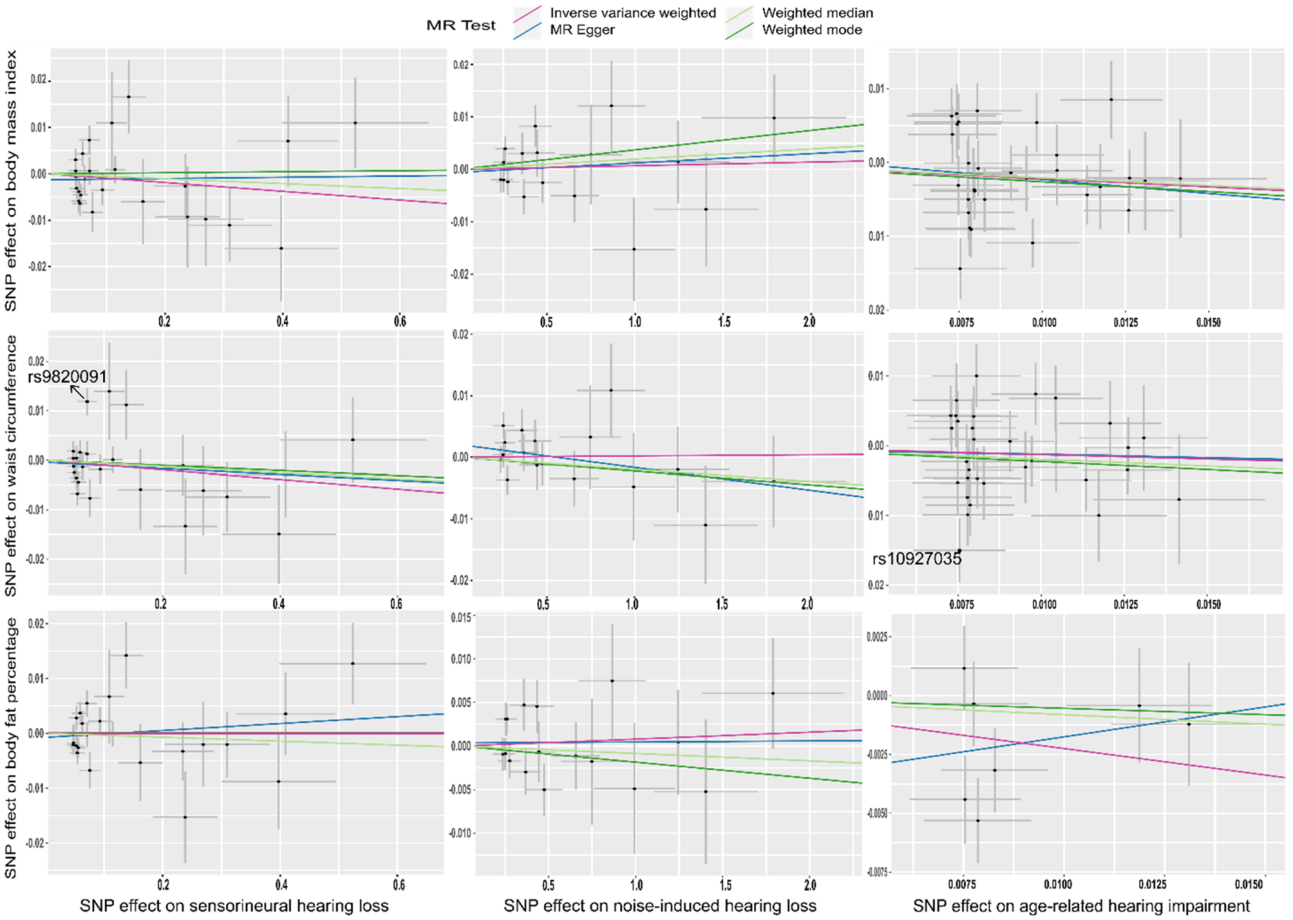
Scatter plots showing Mendelian randomisation sensitivity analysis estimates for the reverse two-sample Mendelian randomisation. *MR-Egger* Mendelian randomisation Egger. Outlier instrumental variables (IVs) detected by MR-PRESSO are labelled with the rsid.

The directions of estimates from sensitivity analyses were consistent with the IVW in the analysis between ARHI liability and BMI (Fig. 4, Supplementary Fig. 2, Supplementary Table 6). We saw heterogeneity in the association (Cochran's Q = 60.44 on 31 degrees of freedom, P = 0.00, I^2^ = 48.7%). The funnel plot of individual SNP showed a relatively symmetrical distribution of SNP effects around the effect estimates, suggesting balanced pleiotropy (Supplementary Fig. 3). The MR-Egger intercept suggested little evidence of directional pleiotropy (Intercept=0.00, P=0.78). We also conducted a leave-one-out analysis (Supplementary Fig. 4) and found no evidence of a single SNP driving the results. The results from the weighted median and weighted mode method were consistent with the IVW in the analysis between ARHI liability and BFP. We did not find evidence of heterogeneity (Cochran’s Q = 10.93 on 6 degrees of freedom, P = 0.09, I^2^ = 45.1%) and directional pleiotropy from the MR-Egger (Intercept = 0.00, P = 0.43).

## Discussion

The present study investigated the causal association between body constitution traits and hearing loss, employing a range of two-sample Mendelian randomisation analyses and complementary sensitivity analyses. Our findings suggest that the commonly positive association between body constitution and hearing loss in observational studies may not correspond to a causal risk-increasing effect. We found evidence that ARHI is negatively associated with BMI and BFP in the reverse MR analyses.

### *FTO* variants may associate with a reduced risk of age-related hearing impairment

Colocalisation analysis suggested that both BMI and ARHI shared the same causal variant, rs1558902, within the *FTO* locus. Based on the well-documented association between FTO variants and BMI^33^, one may favour a scenario in which BMI could influence ARHI. However, there exists a similar probability for the scenarios where ARHI influences BMI or for horizontal pleiotropy in which BMI and AHRI shared a causal variant (Supplementary Fig. 5). The direction of association was the opposite of our hypothesis for a positive association. Our result suggested that people carrying the *FTO* gene variant rs1558902 could be protected from ARHI. Unfortunately, the mechanism behind this association is still unknown and lacks investigation. A joint-GWAS analysis followed by enrichment on hearing- and body constitution-related traits could be a complementary approach. It could provide greater insight into shared biological pathways. Future study is needed to untangle the complexity of underlying mechanisms from a comorbidity/multimorbidity point of view.

### Interpretation of the evidence for a forward causation from body constitution influencing hearing loss outcomes

According to the current literature from observational studies and biological plausibility, we hypothesised that increased BMI, WC and BFP could cause hearing disorders. We found a positive association between BFP and ARHI in the IVW analysis, but the sensitivity analyses suggested the association may be driven by horizontal pleiotropy. Furthermore, the IVs for BFP only explained 0.73% of the variance in ARHI and weak instruments from 70% sample overlap between the two datasets bias the results in the direction of observational association. Overall, we did not find evidence to support such causal effects on either SNHL, NIHL or ARHI, suggesting that residual confounding is likely to explain the previous observations. The previous study also suggested that excess adiposity could alter hair cells in the cochlea of the inner ear^34^. Nonetheless, the use of proxies for fat distribution, such as WC and BFP, did not improve the causal inference of any of the hearing loss phenotypes.

### Interpretation of the evidence for a reverse causation from hearing loss liability influencing body constitution

In the present study, the reverse MR analyses showed little evidence to support a causal effect of SNHL and NIHL on obesity-related outcomes. The sample sizes of GWASes were large and the width of confidence intervals of effect sizes was small, suggesting enough power to detect associations. It should be noted that because SNHL and NIHL are rare outcomes in the Finngen data (7.64% and 0.26%, respectively), we relaxed the p-value threshold from them to increase the number of available IVs. However, the prevalence of NIHL was much higher in the US general population, around 18% of adults aged 20–69 years^35^. Therefore, the result from Finngen may not be very representative of the general population.

### Age-related hearing impairment may reduce body mass index

Our analysis, suggesting genetic evidence that liability to ARHI could be linked with lower BMI and BFP, was supported by the MR sensitivity analyses. This differs from our original hypothesis that hearing loss-related traits would increase body constitution pertaining to a GWAS study made in European participants^40^. It is however consistent with observations from a recent large study conducted in a cohort from the Republic of Korea (2009–2016) reporting a negative association between hearing loss and BMI^13^. There are unfortunately some important ethnic differences between our source data (European ancestors) use in the reverse MR and the aforementioned observation to corroborate evidence.

**Figure 5:**
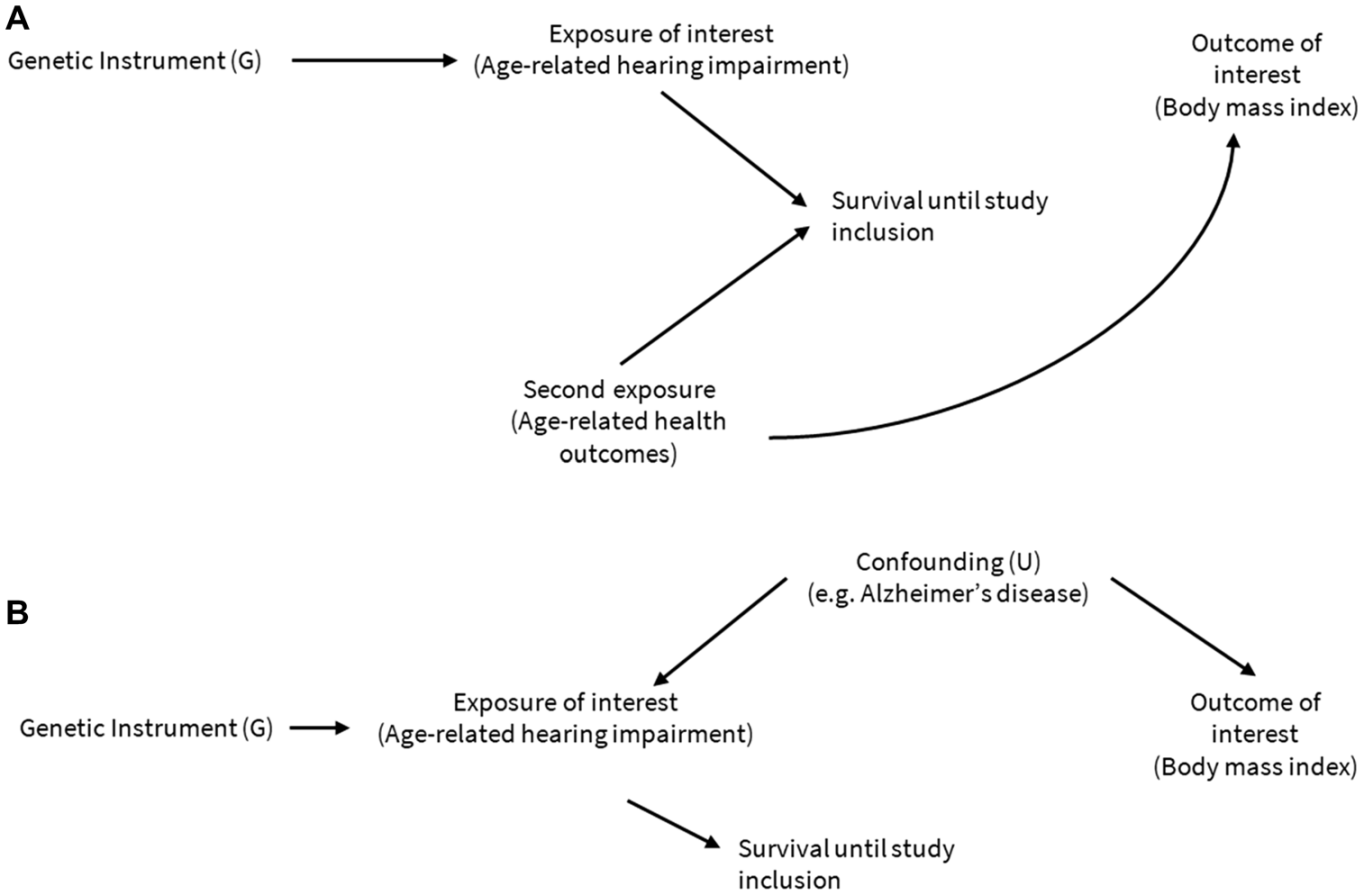
The potential mechanisms linking age-related hearing impairment (ARHI) and body mass index (BMI). For two exposures increasing the risk of death, conditioning on survival may induce an association between the previously uncorrelated ARHI (and its genetic proxy G) and age-related health outcomes (panel A). Additionally, conditioning on survival may induce an association between the genetic instrument G and any confounders U (e.g., Alzheimer’s disease) of the ARHI–BMI association (panel B), even in the absence of second exposure: age-related health outcomes.

Furthermore, we cannot rule out a small chance of selection and survival bias as ARHI is indeed age-related. This pertains to a population that must age. On the other hand, ageing can be associated with lowering of BMI through two mechanisms: (i) people with low BMI or BFP live older and (ii) ageing after a certain age is associated with natural lowering of BMI and fat mass (*i.e.,* sarcopenia). In a two-sample MR setting, when the true effect of an exposure on an outcome is null or >0, the association become nominally negative due to population selection on higher ages and bias to instrumental variable estimators^36^. Figure 5 included the survival factor^36^ and Alzheimer’s disease (AD) as a potential confounder, accounting for the potential spurious negative association. Two MR studies^37,38^ suggested ARHI and weight loss were early manifestations of AD, supporting the notion that the association between ARHI and BMI could be confounded by AD liability. However, there is no data-driven approach conducted to provide evidence to support the notion. ARHI and BMI are in complex and dynamic causal pathways, and further work is needed to understand these mechanisms using MR with mediation analysis.

### Limitations of the study

Some limitations warrant mention. Hearing loss is only affected by large changes in body constitution that may lead to insufficient power for forward MR due to typically low effect sizes of genetic variants. While MR is more robust to unmeasured confounding than conventional epidemiological methods, our results could have been biased by unobserved environmental confounders^39^. For instance, lifestyle-related factors, e.g., diet, drinking and smoking could be on the pathway from ARHI to BMI. If the ARHI SNPs affect these confounders independently, then the assumptions of MR are violated.

An analysis of the relationship between age and body constitution would have allowed estimating the role of declining BMI and BFP as a reflection of population aging leading to ARHI. Subgroup analyses separately for bilateral and unilateral hearing loss would have achieved greater homogeneity, and consequently more accurate results. To the best of our knowledge, such data is unfortunately lacking and commend future investigations. Our MR studies so far have only investigated the linear effects in the general population. Future studies may be designed to investigate the causal role of hearing loss in subpopulations in the state of being underweight, overweight or obese (non-linear effects).

## Conclusion

In conclusion, we found no evidence to support a causal effect of body constitution on hearing loss in our forward MR analyses. Instead, the colocalisation analysis suggested that the *FTO* variant is a common cause of high BMI and low risk of ARHI. In the reverse MR, we found evidence for a causal association between ARHI and lower BMI and BFP. Our results therefore suggest that the mediation of hearing functions is not especially strong for the association between noise exposure and body constitution outcomes.

### Supplementary Information

Supplementary Information 1.

Supplementary Information 2.

## Data Availability

The summary statistics of GWAS used in current study is publicly available in GIANT consortium (https://portals.broadinstitute.org/collaboration/giant/index.php/GIANT_consortium_data_files), Finngen (https://finngen.gitbook.io/documentation/v/r6/data-description#summary-association-statistics) and UK Biobank (https://docs.google.com/spreadsheets/d/1kvPoupSzsSFBNSztMzl04xMoSC3Kcx3CrjVf4yBmESU/edit#gid=227859291). The genetic instrumental variables used in the analyses are presented in Supplementary spreadsheets.
