# Supplementary material for: A bidirectional Mendelian randomisation study to evaluate the relationship between body constitution and hearing loss
Source: Sci Rep. 2023 Oct 27;13:18434. doi: 10.1038/s41598-023-44735-x (PMC10611773; doi:10.1038/s41598-023-44735-x)
Supplement: Supplementary file 1 — Supplementary Information 1. [file 41598_2023_44735_MOESM1_ESM.pdf]

Supplements

A bidirectional Mendelian randomisation study to evaluate the relationship between body constitution and hearing loss

Yiyan He, Ville Karhunen, Anna Pulakka, Marko Kantomaa, Sylvain Sebert

Contents

Supplementary Table 1. Numbers of SNPs used as genetic instrumental variables and F statistics for each pair of exposure and outcome in IVW, MR-Egger, weighted median and weighted mode MR analyses 2

Supplementary Table 2. Results of colocalisation analysis 2

Supplementary Table 3. Effect estimates from forward MR analyses for body mass index, waist circumference, and body fat percentage with various hearing loss diseases 3

Supplementary Table 4. Effect estimates from forward MR sensitivity analyses for adult body mass index, waist circumference, and body fat percentage with various hearing loss diseases 4

Supplementary Table 5. Effect estimates from reverse MR analyses for sensorineural hearing loss, noise-induced hearing loss and age-related hearing impairment with body constitution 6

Supplementary Table 6. Effect estimates from reverse MR sensitivity analyses for sensorineural hearing loss, noise-induced hearing loss and age-related hearing impairment with body constitution 7

Supplementary Figure 1 Forest plot showing the complementary Mendelian randomization estimates for the association between genetically proxied body constitution and the risk of hearing loss 9

Supplementary Figure 2 Forest plot showing the complementary Mendelian randomization estimates for the association between genetically proxied hearing loss traits and body constitution 10

Supplementary Figure 3 Funnel plot for MR analysis of age-related hearing impairment and body mass index. 11

Supplementary Figure 4 Leave-one-out analysis for age-related hearing impairment liability and body mass index 12

Supplementary Figure 5 Three scenarios of shared causal variant (colocalisation) between body mass index and age-related hearing impairment 13

Supplementary Table 1. Numbers of SNPs used as genetic instrumental variables and F statistics for each pair of exposure and outcome in IVW, MR-Egger, weighted median and weighted mode MR analyses

Outcomes	Body mass index			Waist circumference			Body Fat percentage		
	pval	N_SNPs	Median *F* (range)	pval	N_SNPs	Median *F* (range)	pval	N_SNPs	Median F (range)
Sensorineural hearing loss	5E-08	89	62 (30–294)	5E-08	72	53 (30–223)	5E-08	85	53 (30–180)
Noise-induced hearing loss	5E-08	89	62 (30–294)	5E-08	72	53 (30–223)	5E-08	85	53 (30–180)
Age-related hearing impairment	5E-08	68	67 (29–688)	5E-08	41	60 (29–447)	5E-08	87	52 (30–180)
Outcomes	Sensorineural hearing loss			Noise-induced hearing loss			Age-related hearing impairment		
	pval	N_SNPs	Median F (range)	pval	N_SNPs	Median F (range)	pval	N_SNPs	Median F (range)
Body mass index	5E-05	26	20 (17–45)	5E-05	17	19 (18–23)	5E-08	32	42 (30–91)
Waist circumference	5E-05	25	20 (17–45)	5E-05	17	19 (18–23)	5E-08	32	42 (30–91)
Body Fat percentage	5E-05	25	20 (17–45)	5E-05	17	19 (18–23)	5E-08	7	37 (30–45)

Abbreviations: pval P value threshold used for selecting instrumental variants, SNPs single nucleotide polymorphisms, MR Mendelian Randomisation

Supplementary Table 2. Results of colocalisation analysis

Models	Posterior Probabilities
H0	0.00000
H1	0.00006
H2	0.00000
H3	0.07980
H4	0.92000

Supplementary Table 3. Effect estimates from forward MR analyses for body mass index, waist circumference, and body fat percentage with various hearing loss diseases

Exposure	Outcome	Method	OR	lo_ci	up_ci	pval	*P*het
Body mass index	Sensorineural hearing loss	Inverse variance weighted	1.01	0.88	1.16	0.923	0.20
Body mass index	Noise-induced hearing loss	Inverse variance weighted	1.44	0.76	2.73	0.268	0.85
Body mass index	Age-related hearing impairment	Inverse variance weighted	0.99	0.97	1.00	0.108	0.00
Waist circumference	Sensorineural hearing loss	Inverse variance weighted	1.00	0.84	1.20	0.960	0.54
Waist circumference	Noise-induced hearing loss	Inverse variance weighted	0.89	0.37	2.15	0.802	0.46
Waist circumference	Age-related hearing impairment	Inverse variance weighted	0.98	0.96	1.00	0.087	0.00
Body fat percentage	Sensorineural hearing loss	Inverse variance weighted	0.85	0.69	1.06	0.146	0.06
Body fat percentage	Noise-induced hearing loss	Inverse variance weighted	1.40	0.56	3.51	0.477	0.67
Body fat percentage	Age-related hearing impairment	Inverse variance weighted	1.04	1.01	1.06	0.003	0.02

Phet: P-values of Chi-square Q test for heterogeneity. Abbreviations: MR Mendelian Randomisation, OR odds ratio, 95 % CI confidence intervals, p/pval P value, het heterogenity

Supplementary Table 4. Effect estimates from forward MR sensitivity analyses for adult body mass index, waist circumference, and body fat percentage with various hearing loss diseases

Exposure	Outcome	Method	SNPs	OR	or_lci95	or_uci95	pval	int	p_pleiotropy
Body mass index	Sensorineural hearing loss	MR Egger	89	1.30917	0.88630	1.93381	0.17939	-0.00589	0.16157
Body mass index	Sensorineural hearing loss	Inverse variance weighted	89	1.00694	0.87537	1.15828	0.92292	NA	NA
Body mass index	Sensorineural hearing loss	Weighted mode	89	0.93768	0.67361	1.30528	0.70391	NA	NA
Body mass index	Sensorineural hearing loss	Weighted median	89	0.97385	0.79387	1.19463	0.79937	NA	NA
Body mass index	Noise-induced hearing loss	MR Egger	89	0.57076	0.09429	3.45490	0.54317	0.02072	0.28469
Body mass index	Noise-induced hearing loss	Inverse variance weighted	89	1.43762	0.75598	2.73386	0.26832	NA	NA
Body mass index	Noise-induced hearing loss	Weighted mode	89	0.83938	0.14699	4.79309	0.84430	NA	NA
Body mass index	Noise-induced hearing loss	Weighted median	89	1.49129	0.55716	3.99156	0.42626	NA	NA
Body mass index	Age-related hearing impairment	MR Egger	68	0.93964	0.90697	0.97349	0.00099	0.00159	0.00363
Body mass index	Age-related hearing impairment	Inverse variance weighted	68	0.98762	0.97274	1.00273	0.10790	NA	NA
Body mass index	Age-related hearing impairment	Weighted mode	68	0.98785	0.93632	1.04222	0.65617	NA	NA
Body mass index	Age-related hearing impairment	Weighted median	68	0.98795	0.96782	1.00850	0.24832	NA	NA
Body mass index	Age-related hearing impairment	MR-PRESSO	66	0.99783	0.98464	1.01120	0.75028	NA	NA
Waist circumference	Sensorineural hearing loss	MR Egger	72	1.08024	0.60848	1.91777	0.79289	-0.00133	0.79482
Waist circumference	Sensorineural hearing loss	Inverse variance weighted	72	1.00459	0.83942	1.20225	0.96017	NA	NA
Waist circumference	Sensorineural hearing loss	Weighted mode	72	0.91174	0.56758	1.46458	0.70352	NA	NA
Waist circumference	Sensorineural hearing loss	Weighted median	72	0.96823	0.73781	1.27061	0.81589	NA	NA
Waist circumference	Noise-induced hearing loss	MR Egger	72	0.95754	0.05686	16.12479	0.97606	-0.00126	0.96003
Waist circumference	Noise-induced hearing loss	Inverse variance weighted	72	0.89385	0.37174	2.14932	0.80206	NA	NA
Waist circumference	Noise-induced hearing loss	Weighted mode	72	0.68089	0.04909	9.44430	0.77536	NA	NA
Waist circumference	Noise-induced hearing loss	Weighted median	72	0.93072	0.24188	3.58130	0.91683	NA	NA

Waist circumference	Age-related hearing impairment	MR Egger	41	0.89027	0.84653	0.93628	0.00006	0.00308	0.00023
Waist circumference	Age-related hearing impairment	Inverse variance weighted	41	0.98070	0.95908	1.00281	0.08664	NA	NA
Waist circumference	Age-related hearing impairment	Weighted mode	41	0.98196	0.91490	1.05393	0.61667	NA	NA
Waist circumference	Age-related hearing impairment	Weighted median	41	0.98436	0.95902	1.01037	0.23617	NA	NA
Waist circumference	Age-related hearing impairment	MR-PRESSO	39	0.99461	0.97559	1.01401	0.58672	NA	NA
Body fat percentage	Sensorineural hearing loss	MR Egger	85	1.31098	0.63659	2.69982	0.46462	-0.00645	0.22777
Body fat percentage	Sensorineural hearing loss	Inverse variance weighted	85	0.85431	0.69108	1.05609	0.14551	NA	NA
Body fat percentage	Sensorineural hearing loss	Weighted mode	85	0.94407	0.54408	1.63813	0.83831	NA	NA
Body fat percentage	Sensorineural hearing loss	Weighted median	85	0.86983	0.65358	1.15763	0.33893	NA	NA
Body fat percentage	Noise-induced hearing loss	MR Egger	85	0.94586	0.04074	21.95733	0.97241	0.00588	0.80005
Body fat percentage	Noise-induced hearing loss	Inverse variance weighted	85	1.39676	0.55626	3.50725	0.47685	NA	NA
Body fat percentage	Noise-induced hearing loss	Weighted mode	85	1.88896	0.13125	27.18679	0.64137	NA	NA
Body fat percentage	Noise-induced hearing loss	Weighted median	85	1.74865	0.48888	6.25464	0.39010	NA	NA
Body fat percentage	Age-related hearing impairment	MR Egger	87	0.96954	0.89287	1.05279	0.46378	0.00103	0.09418
Body fat percentage	Age-related hearing impairment	Inverse variance weighted	87	1.03769	1.01233	1.06369	0.00338	NA	NA
Body fat percentage	Age-related hearing impairment	Weighted mode	87	0.96885	0.88805	1.05701	0.47831	NA	NA
Body fat percentage	Age-related hearing impairment	Weighted median	87	1.01730	0.98343	1.05233	0.32080	NA	NA

NA indicate not applicable. OR: odds ratio. Int: the intercept of MR-Egger regression; MR-PRESSO: Mendelian Randomization Pleiotropy RESidual Sum and Outlier. SNPs single nucleotide polymorphisms.

Supplementary Table 5. Effect estimates from reverse MR analyses for sensorineural hearing loss, noise-induced hearing loss and age-related hearing impairment with body constitution

Exposure	Outcome	Method	beta	lo_ci	up_ci	pval	*P*het
Sensorineural hearing loss	Body mass index	Inverse variance weighted	-0.009	-0.029	0.010	0.351	0.006
Sensorineural hearing loss	Waist circumference	Inverse variance weighted	-0.010	-0.028	0.009	0.305	0.001
Sensorineural hearing loss	Body fat percentage	Inverse variance weighted	0.000	-0.014	0.014	0.998	0.027
Noise-induced hearing loss	Body mass index	Inverse variance weighted	0.001	-0.004	0.005	0.766	0.187
Noise-induced hearing loss	Waist circumference	Inverse variance weighted	0.000	-0.003	0.004	0.916	0.436
Noise-induced hearing loss	Body fat percentage	Inverse variance weighted	0.001	-0.002	0.004	0.634	0.295
Age-related hearing impairment	Body mass index	Inverse variance weighted	-0.221	-0.436	-0.007	0.043	0.001
Age-related hearing impairment	Waist circumference	Inverse variance weighted	-0.129	-0.346	0.088	0.245	0.031
Age-related hearing impairment	Body fat percentage	Inverse variance weighted	-0.225	-0.447	-0.003	0.047	0.090

P_{het}: P-values of Chi-square Q test for heterogeneity. Abbreviations: MR Mendelian Randomisation, 95 % CI confidence intervals

Supplementary Table 6. Effect estimates from reverse MR sensitivity analyses for sensorineural hearing loss, noise-induced hearing loss and age-related hearing impairment with body constitution

Exposure	Outcome	Method	SNPs	beta	lo_ci	up_ci	pval	int	p_pleiotropy
Sensorineural hearing loss	Body mass index	MR Egger	25	0.00134	-0.03036	0.03303	0.93492	-0.00128	0.40029
Sensorineural hearing loss	Body mass index	Inverse variance weighted	25	-0.00943	-0.02928	0.01041	0.35149	NA	NA
Sensorineural hearing loss	Body mass index	Weighted mode	25	0.00114	-0.02576	0.02803	0.93475	NA	NA
Sensorineural hearing loss	Body mass index	Weighted median	25	-0.00529	-0.02780	0.01721	0.64490	NA	NA
Sensorineural hearing loss	Waist circumference	MR Egger	25	-0.00609	-0.03619	0.02401	0.69519	-0.00043	0.76308
Sensorineural hearing loss	Waist circumference	Inverse variance weighted	25	-0.00973	-0.02832	0.00886	0.30477	NA	NA
Sensorineural hearing loss	Waist circumference	Weighted mode	25	-0.00516	-0.02607	0.01575	0.63274	NA	NA
Sensorineural hearing loss	Waist circumference	Weighted median	25	-0.00628	-0.02634	0.01379	0.53966	NA	NA
Sensorineural hearing loss	Waist circumference	MR-PRESSO	24	-0.01479	-0.02951	-0.00007	0.06097	NA	NA
Sensorineural hearing loss	Body fat percentage	MR Egger	25	0.00632	-0.01639	0.02902	0.59076	-0.00075	0.49077
Sensorineural hearing loss	Body fat percentage	Inverse variance weighted	25	0.00001	-0.01413	0.01416	0.99841	NA	NA
Sensorineural hearing loss	Body fat percentage	Weighted mode	25	0.00009	-0.02136	0.02153	0.99374	NA	NA
Sensorineural hearing loss	Body fat percentage	Weighted median	25	-0.00359	-0.02055	0.01337	0.67822	NA	NA
Noise-induced hearing loss	Body mass index	MR Egger	17	0.00178	-0.00665	0.01022	0.68404	-0.00058	0.76381
Noise-induced hearing loss	Body mass index	Inverse variance weighted	17	0.00069	-0.00385	0.00523	0.76601	NA	NA
Noise-induced hearing loss	Body mass index	Weighted mode	17	0.00372	-0.00478	0.01222	0.40389	NA	NA
Noise-induced hearing loss	Body mass index	Weighted median	17	0.00195	-0.00421	0.00810	0.53588	NA	NA
Noise-induced hearing loss	Waist circumference	MR Egger	17	-0.00371	-0.01007	0.00266	0.27153	0.00205	0.16939
Noise-induced hearing loss	Waist circumference	Inverse variance weighted	17	0.00019	-0.00337	0.00375	0.91551	NA	NA
Noise-induced hearing loss	Waist circumference	Weighted mode	17	-0.00225	-0.00898	0.00448	0.52206	NA	NA
Noise-induced hearing loss	Waist circumference	Weighted median	17	-0.00196	-0.00702	0.00309	0.44627	NA	NA
Noise-induced hearing loss	Body fat percentage	MR Egger	17	0.00012	-0.00596	0.00620	0.97056	0.00036	0.79574
Noise-induced hearing loss	Body fat percentage	Inverse variance weighted	17	0.00080	-0.00248	0.00407	0.63370	NA	NA

Noise-induced hearing loss	Body fat percentage	Weighted mode	17	-0.00185	-0.00801	0.00431	0.56448	NA	NA
Noise-induced hearing loss	Body fat percentage	Weighted median	17	-0.00085	-0.00515	0.00345	0.69848	NA	NA
Age-related hearing impairment	Body mass index	MR Egger	32	-0.37731	-1.48376	0.72913	0.50900	0.00145	0.77978
Age-related hearing impairment	Body mass index	Inverse variance weighted	32	-0.22116	-0.43569	-0.00664	0.04332	NA	NA
Age-related hearing impairment	Body mass index	Weighted mode	32	-0.26290	-0.64600	0.12021	0.18838	NA	NA
Age-related hearing impairment	Body mass index	Weighted median	32	-0.20823	-0.44418	0.02771	0.08367	NA	NA
Age-related hearing impairment	Waist circumference	MR Egger	32	-0.09973	-1.22025	1.02078	0.86268	-0.00027	0.95899
Age-related hearing impairment	Waist circumference	Inverse variance weighted	32	-0.12880	-0.34584	0.08824	0.24477	NA	NA
Age-related hearing impairment	Waist circumference	Weighted mode	32	-0.22868	-0.63148	0.17411	0.27436	NA	NA
Age-related hearing impairment	Waist circumference	Weighted median	32	-0.19574	-0.45607	0.06458	0.14055	NA	NA
Age-related hearing impairment	Waist circumference	MR-PRESSO	31	-0.08771	-0.28708	0.11166	0.39538	NA	NA
Age-related hearing impairment	Body fat percentage	MR Egger	7	0.25397	-0.86211	1.37005	0.67424	-0.00429	0.42955
Age-related hearing impairment	Body fat percentage	Inverse variance weighted	7	-0.22497	-0.44717	-0.00276	0.04722	NA	NA
Age-related hearing impairment	Body fat percentage	Weighted mode	7	-0.05402	-0.38922	0.28119	0.76283	NA	NA
Age-related hearing impairment	Body fat percentage	Weighted median	7	-0.08103	-0.31527	0.15320	0.49773	NA	NA

NA indicate not applicable. Int: the intercept of MR-Egger regression; ci: confidence interval (95%); MR-PRESSO: Mendelian Randomization Pleiotropy RESidual Sum and Outlier. SNPs single nucleotide polymorphisms

Supplementary Figure 1 Forest plot showing the complementary Mendelian randomization estimates for the association between genetically proxied body constitution and the risk of hearing loss

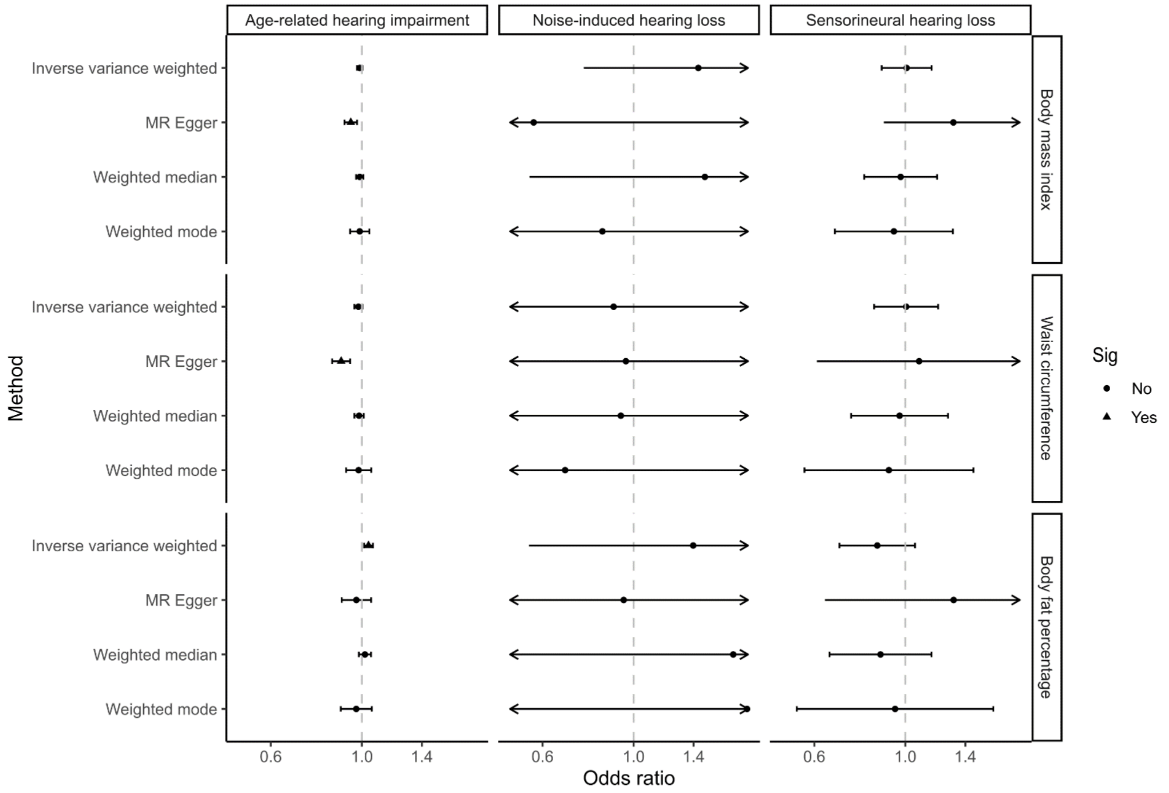

MR-Egger: Mendelian randomisation Egger. Sig: significance (p-value < 0.5). Odds ratio (OR) with 95% confidence interval. Arrows indicate 0.3>OR or OR>2.0

Supplementary Figure 2 Forest plot showing the complementary Mendelian randomization estimates for the association between genetically proxied hearing loss traits and body constitution

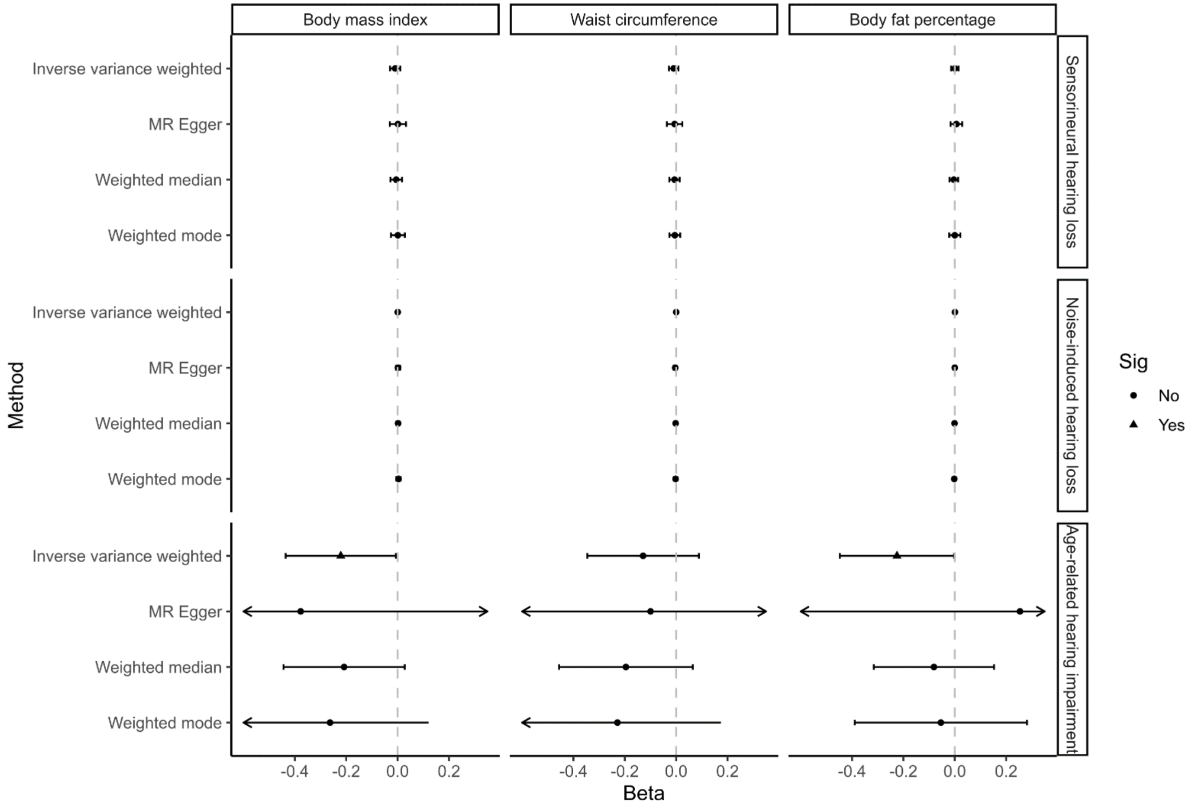

MR-Egger: Mendelian randomisation Egger. Sig: significance (p-value < 0.5). Effect sizes with 95% confidence interval. Arrows indicate -0.6>beta or beta>0.35

Supplementary Figure 3 Funnel plot for MR analysis of age-related hearing impairment and body mass index.

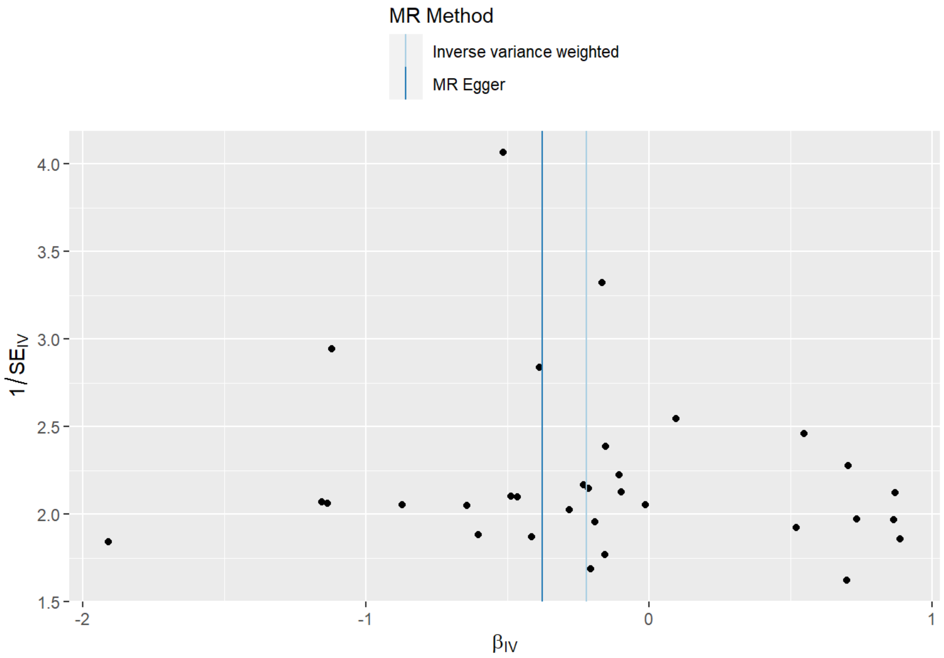

MR-Egger: Mendelian randomisation Egger

Supplementary Figure 4 Leave-one-out analysis for age-related hearing impairment liability and body mass index

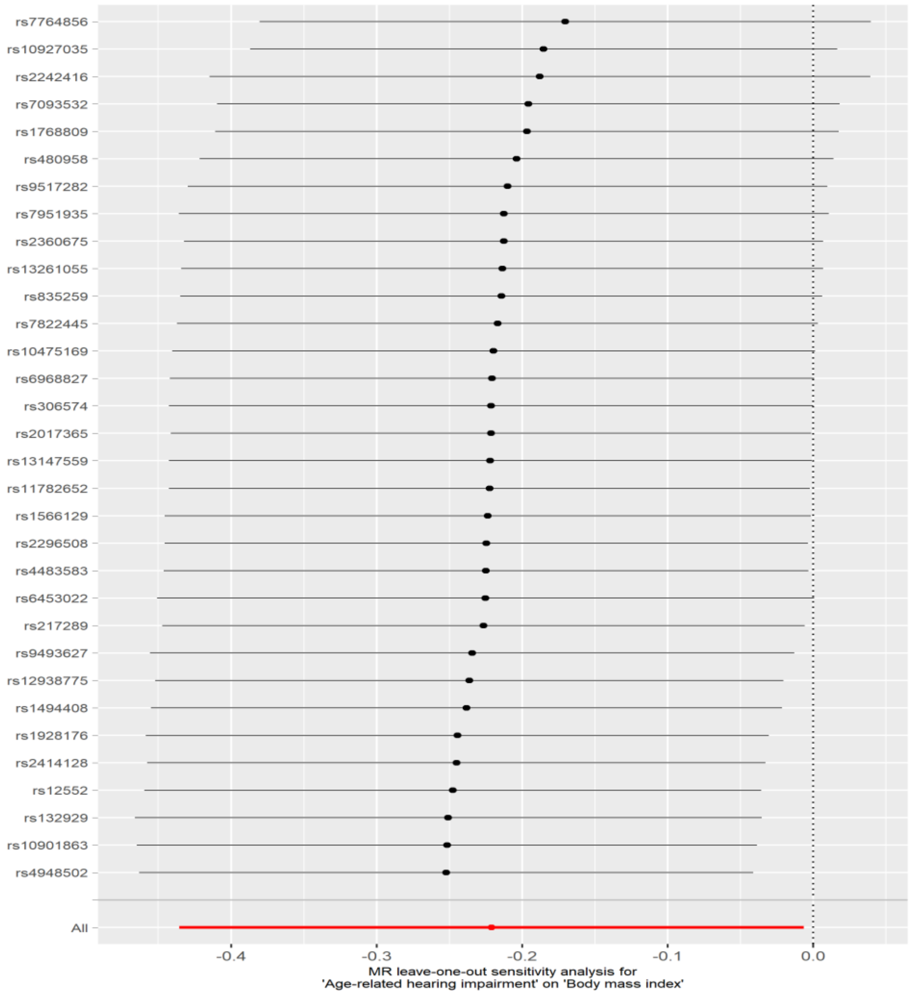

Effect size (x-axis) after leaving one instrumental variant (y-axis) out using inverse variance weighted method

Supplementary Figure 5 Three scenarios of shared causal variant (colocalisation) between body mass index and age-related hearing impairment

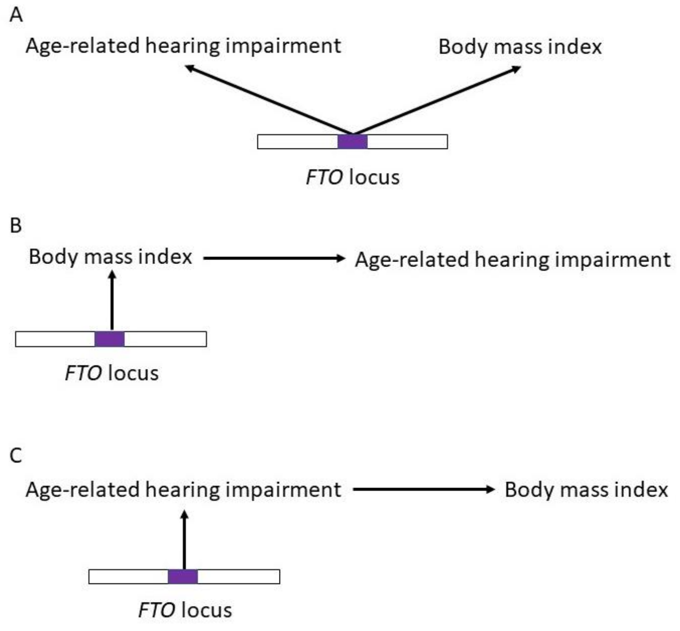

There are three possible scenarios of colocalisation between body mass index (BMI) and age-related hearing impairment (ARHI).

Panel A: two unrelated traits with a shared causal variant.

Panel B and C: two traits with a shared causal variant where the first trait influences the second trait, which are BMI influences ARHI and ARHI influences BMI.
